# SD118-Xanthocillin X (1), a Novel Marine Agent Extracted from *Penicillium commune*, Induces Autophagy through the Inhibition of the MEK/ERK Pathway

**DOI:** 10.3390/md10061345

**Published:** 2012-06-11

**Authors:** Ying Zhao, Huan Chen, Zhuo Shang, Binghua Jiao, Bin Yuan, Weizhang Sun, Bingui Wang, Mingyong Miao, Caiguo Huang

**Affiliations:** 1 Department of Biochemistry and Molecular Biology, Second Military Medical University, 800 Xiangyin Road, Shanghai 200433, China; Email: neely.zhao@gmail.com (Y.Z.); chenhuansym@hotmail.com (H.C.); jiaobh@uninet.cn (B.J.); yuanbin7882@sohu.com (B.Y.); 2 Key Laboratory of Experimental Marine Biology, Institute of Oceanology, Chinese Academy of Sciences, Qingdao 266071, China; Email: alexsz1985@gmail.com; 3 PET (Positron Emission Computed Tomography) Center, General Hospital of Chengdu Military Command, Chengdu, Sichuan 610083, China; Email: sunwz@msn.com

**Keywords:** SD118-xanthocillin X (**1**), autophagy, apoptosis, ERK, Beclin 1

## Abstract

A compound named SD118-xanthocillin X (**1**) (C_18_H_12_N_2_O_2_), isolated from *Penicillium commune* in a deep-sea sediment sample, has been shown to inhibit the growth of several cancer cell lines *in vitro*. In the present study, we employed a growth inhibition assay and apoptotic analysis to identify the biological effect and detailed mechanism of SD118-xanthocillin X (**1**) in human hepatocellular carcinoma (HepG2) cells. SD118-xanthocillin X (**1**) demonstrated a concentration-dependent inhibitory effect on the growth of HepG2 cells and caused slight cellular apoptosis and significantly induced autophagy. Autophagy was detected as early as 12 h by the conversion of microtubule-associated protein 1 light chain 3 (LC3-I) to LC3-II, following cleavage and lipid addition to LC3-I. The pharmacological autophagy inhibitor 3-methyladenine largely attenuates the growth inhibition and autophagic effect of SD118-xanthocillin X (**1**) in HepG2 cells. Our data also indicated that the autophagic effect of SD118-xanthocillin X (**1**) occurs via the down-regulation of the MEK/ERK signaling pathway and the up-regulated class III PI3K/Beclin 1 signaling pathway.

## 1. Introduction

A novel “marine source” of anticancer preclinical and clinical agents has emerged through persistent efforts over the last decade to explore the abundant chemical diversity offered by marine organisms (e.g., Curacin A; Salinosporamide A; Vitilevuamide; Ecteinascidin 743). Marine life can produce a variety of compounds in response to intense competition, termed secondary metabolites, including such chemical classes as terpenoids, alkaloids, polyketides and peptides and a multitude of mixed-biogenesis metabolites. In the past decade, there has been a dramatic increase in the number of lead preclinical anticancer compounds from diverse marine organisms entering clinical trials [1]. One of the most promising areas is cancer therapy. Currently, there are two drugs approved by the Food and Drug Administration (FDA) and European Agency for the Evaluation of Medicinal Products (EMEA) for cancer treatment, Cytarabine (Cytosar-U1^®^) and Eribulin (E7389 or Halaven^®^) [2]. Eribulin (E7389) is an inhibitor of microtubule dynamics: it binds to tubulin and inhibits microtubule polymerization [3,4] and exerts its anticancer effects by triggering apoptosis in cancer cells following the blockage of mitosis [5,6]. As with many anticancer drugs, the mode of action of anti-microtubule agents involves the induction of programmed cell death (PCD).

Apoptosis (Type I PCD) is characterized by a rounding of the cell shape, a retraction of pseudopodia, a reduction of the cellular volume, chromatin condensation (pyknosis) and nuclear fragmentation (karyorrhexis). In recent years, it became evident that other forms of cell death are also programmed; among them, autophagic cell death (ACD; Type II PCD) is now recognized as an important process involved in different human pathologies, such as disease, aging and cancer [7,8]. Recent studies have suggested that autophagy is important in the regulation of cancer development and progression, particularly in determining the response of tumor cells to anticancer therapy [9]. In fact, the induction of autophagy has been observed in response to several anticancer drugs, such as temozolomide and dexamethasone, and autophagy plays a key role in cancer therapy by triggering a non-apoptotic cell death program: this type of autophagy is irreversible and is referred to as ACD [10,11]. 

Autophagy is a catabolic process involving the degradation of a cell's own components through the lysosomal machinery [12,13]. During autophagy, the part of the cytoplasm containing long-lived proteins or organelles is surrounded by an isolation membrane, which then closes to form a double-membrane vacuole known as the autophagosome [14]. The formation of autophagosomes is dependent on the induction of several genes, including LC3, Beclin 1 and Bcl-2. 

**Figure 1:**
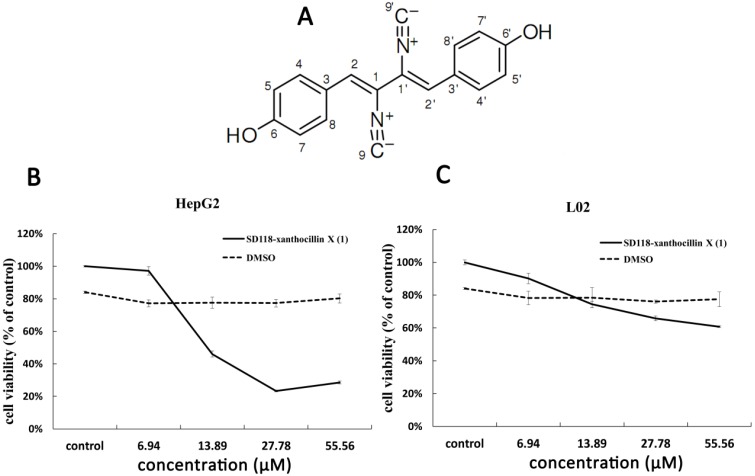
Chemical structure of SD118-xanthocillin X (**1**) and effects of SD118-xanthocillin X (**1**) on cytotoxic activity. (**A**) Chemical structure of SD118-xanthocillin X (**1**); (**B**,**C**) Effects of SD118-xanthocillin X (**1**) on cytotoxic activity of HepG2 cell and normal liver cell line L02. HepG2 cells (**B**) and L02 (**C**) cells were treated with SD118-xanthocillin X (**1**) of 6.9 µM, 13.89 µM, 27.78 µM, 55.56 µM for 48 h, and DMSO was used as control in both groups.

Recently, we identified SD118-xanthocillin X (**1**) (C_18_H_12_N_2_O_2_) (structure shown in Figure 1A), which was obtained from *Penicillium commune*,found in a deep-sea sediment sample, and found that the compound inhibits the growth of several cancer cell lines *in vitro* [15]. The present investigation was designed to characterize the action of SD118-xanthocillin X (**1**) in hepatocellular liver carcinoma cells (HepG2) and to investigate the molecular mechanisms by which SD118-xanthocillin X (**1**) caused cell death. Intriguingly, our study shows that SD118-xanthocillin X (**1**) induced slight cell apoptosis and significant autophagy in HepG2 cells. One proposed downstream effector of ERK involved in the regulation of autophagy is mTOR kinase, which inhibits autophagy by phosphorylating downstream substrates that are possibly analogous to other autophagy associated gene (ATG) products [16,17]. We found that class III PI3K/Beclin 1 and the MEK/ERK signaling pathway are involved in the SD118-xanthocillin X (**1**)-induced autophagy process in HepG2 cells, a process that was partially attenuated by 3-methyladenine. These data reveal that SD118-xanthocillin X (**1**) induced autophagy in HepG2 cells via a non-canonical signaling pathway [17].

## 2. Results and Discussion

### 2.1. Cytotoxic Activity of SD118-Xanthocillin X *(**1**)* in HepG2 Cells

HepG2 cells and a normal cell line L02 (human normal liver cell line cells) were chosen to evaluate the cytotoxic activity of SD118-xanthocillin X (**1**). Cell viability was examined by a formazan-based MTT cell viability assay as described in “Materials and Methods”. A concentration-dependent inhibitory effect by SD118-xanthocillin X (**1**) on cellular growth was observed in the HepG2 cells, with the IC_50_ at the 0–55.56 µM (0–16 μg/mL) dosage being 22.88 ± 4.76 µM (6.59 ± 1.37 μg/mL) after 48 h. These data indicated that SD118-xanthocillin X (**1**) had a significant inhibitory effect on the proliferation of HepG2 cells (Figure 1B). However, we observed that SD118-xanthocillin X (**1**) had no statistically significant effect on normal liver cells (L02 cells) (Figure 1C).

**Figure 2:**
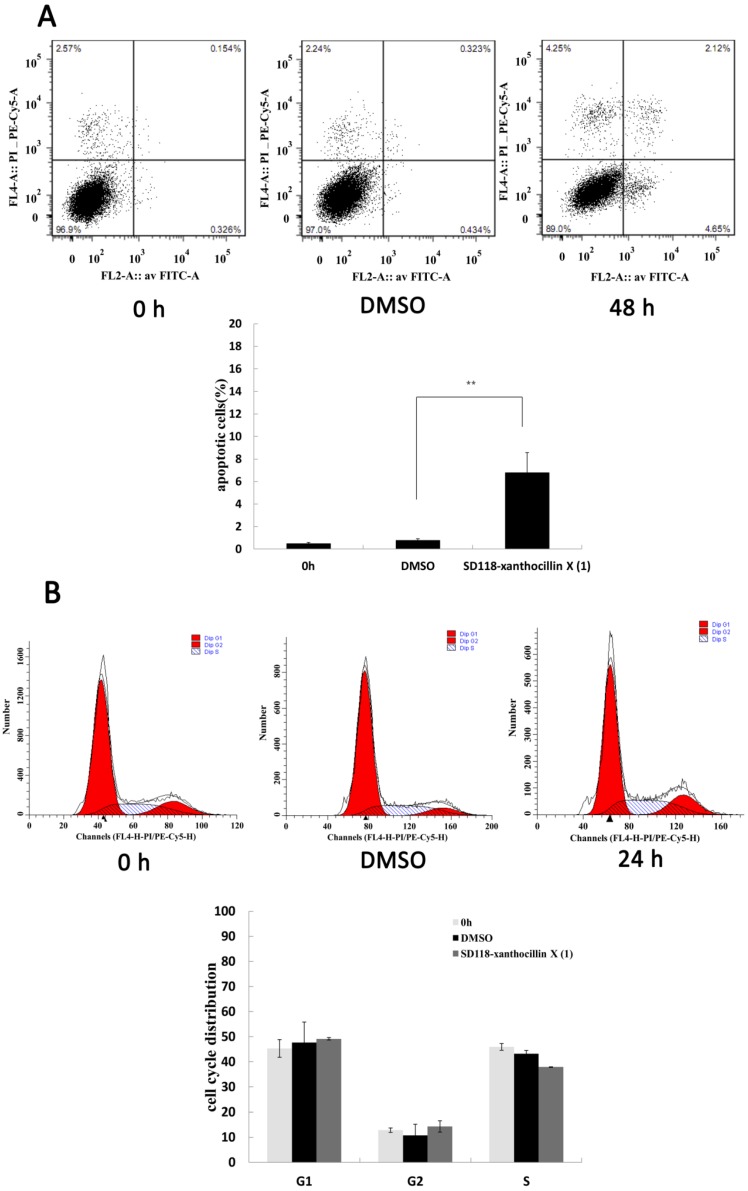
SD 118-2 induces apoptosis and cell cycle in HepG2 cells (**A**,**B**). Flow cytometry analysis of apoptosis (**A**) and cell cycle (**B**). HepG2 cells were treated with 24.3 µM SD118-xanthocillin X (**1**) for indicated times, its negative control was treated with 24.3 µM DMSO. Then cells were harvested and stained with annexin V-FITC-PI or PI. ** *p* < 0.01.

Since cell viability was significantly inhibited by SD118-xanthocillin X (**1**), it was critical to classify which type of cell death was induced in HepG2 cells. An annexin V/PI assay was performed to detect the apoptosis and cell cycle distribution by SD118-xanthocillin X (**1**) treatment. Our data showed that SD118-xanthocillin X (**1**) slightly induced apoptosis (Figure 2A) and did not alter the cell cycle distribution of the HepG2 cells (Figure 2B). To explore the mechanism of the growth inhibitory effect of SD118-xanthocillin X (**1**) further, we assayed its autophagy effect, which has been shown to be a novel response to some anticancer agents that induce autophagy and trigger an autophagic cell death (ACD) response. 

### 2.2. SD118-Xanthocillin X *(**1**)* Induces Apoptosis and Autophagy in HepG2 Cells

**Figure 3:**
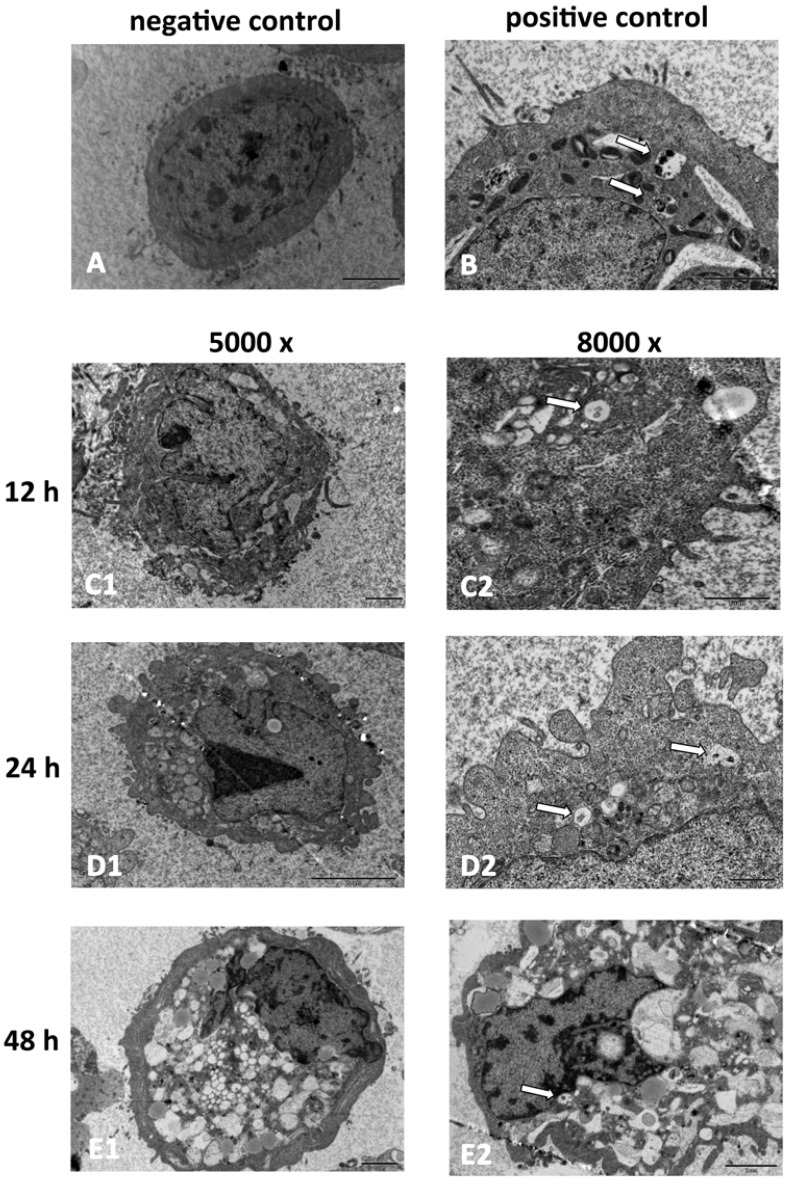
SD118-xanthocillin X (**1**) induces autophagy in HepG2 cells. SD118-xanthocillin X (**1**) induces autophagy in HepG2 cells. Representative TEM photomicrographs of HepG2 cells treated with SD118-xanthocillin X (**1**) for different times. The negative control was treated with 24.3 µM DMSO and positive control was starved by RPMI 1640 medium without serum for 48 h. (**A**) Microvillus and normal nuclear morphologies exhibit in negative control cells; (**B**) Autophagic vacuoles contain remnants of organelles in positive control cells; (**C**) SD118-Xanthocillin X (**1**) treatment for 12 h resulted in the development of autophagic vacuoles (**C1**: 5000×; **C2**: 8000×); (**D**) The autophagic vacuoles show clearly at 24 h following SD118-xanthocillin X (**1**) treatment and organelles were degraded (**D1**: 5000×; **D2**: 8000×); (**E**) The number of vacuoles was increased by SD118-xanthocillin X (**1**) treatment for 48 h (**E1**: 5000×; **E2**: 8000×). Higher magnification showed that autophagic vacuoles (the arrow points) contain remnants of organelles.

Using transmission electron microscopy (TEM), which is the standard method to detect autophagy reliably [12], we observed the formation of autophagosomes in the HepG2 cells after SD118-xanthocillin X (**1**) treatment (Figure 3). As shown in Figure 3, the negative control cells exhibited normal nuclei, with uniform, dispersed chromatin and abundant microvilli on the plasmalemma (Figure 3A). The detection of starvation-induced autophagy was used as a positive control (Figure 3B). In contrast, SD118-xanthocillin X (**1**) treatment for 12 h resulted in indistinguishable organelles and a decrease in microvilli (Figure 3C). After 24 h, the appearance of autophagic vacuoles containing degraded organelles was found (Figure 3D), and the formation of these membranous vacuoles increased gradually over time (Figure 3E). A higher magnification showed that the autophagic vacuoles contained the remnants of cellular organelles. These biological indicators demonstrated that SD118-xanthocillin X (**1**) results in the appearance of autophagosomes in HepG2 cells.

### 2.3. The Expression of Autophagy-Related Genes

LC3 has been shown to be an autophagosomal marker in mammals. LC3 usually exists in two forms: LC3-I (16 kDa) and LC3-II (14 kDa). The amount of LC3-II or the ratio of LC3-II/LC3-I correlates with the status of autophagosomes [18]. Beclin 1 is involved in both signaling pathways that activate autophagy and in the initial step of autophagosomal formation [19,20].

**Figure 4:**
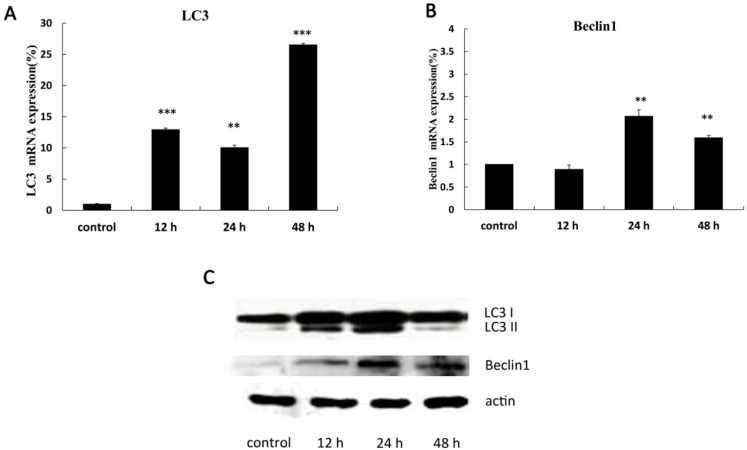
SD118-Xanthocillin X (**1**) induced autophagic gene expression in HepG2 cells (**A**,**B**). SD118-Xanthocillin X (**1**) increased the mRNA expression of LC3 (**A**) and Beclin 1 (**B**). HepG2 cells were treated with 24.3 µM SD118-xanthocillin X (**1**) for indicated times, and relative mRNA expression of autophagic genes was measured by quantitative Real-Time PCR analysis. (** *p* < 0.001, *** *p* < 0.0001) compared to control group;(**C**) HepG2 cells were treated with 24.3 µM SD118-xanthocillin X (**1**) for indicated times. Western blot represents the conversion of LC3-I to LC3-II and time-dependent up-regulation of Beclin 1 following SD118-xanthocillin X (**1**) treatment. Protein contents were normalized with β-actin.

Our results showed that the mRNA expression of the LC3 (Figure 4A) and Beclin 1 (Figure 4B) genes were up-regulated after 12 h of SD118-xanthocillin X (**1**) treatment in a time-dependent manner in HepG2 cells by quantitative real-time PCR. Moreover, western blot analysis confirmed that SD118-xanthocillin X (**1**) altered the expression of autophagy-related genes at the protein level. We examined the expression of Beclin 1 and the conversion of LC3-I to LC3-II in the HepG2 cells after SD118-xanthocillin X (**1**) treatment at different time points; as shown in Figure 4C, the increased expression was time dependent and most notable after 12 h. Thus, the above data indicate that SD118-xanthocillin X (**1**) increased the expression of the autophagy-related genes, LC3, and Beclin 1.

### 2.4. 3-MA Partially Attenuates the Autophagic Effect of SD118-Xanthocillin X *(**1**)*

**Figure 5:**
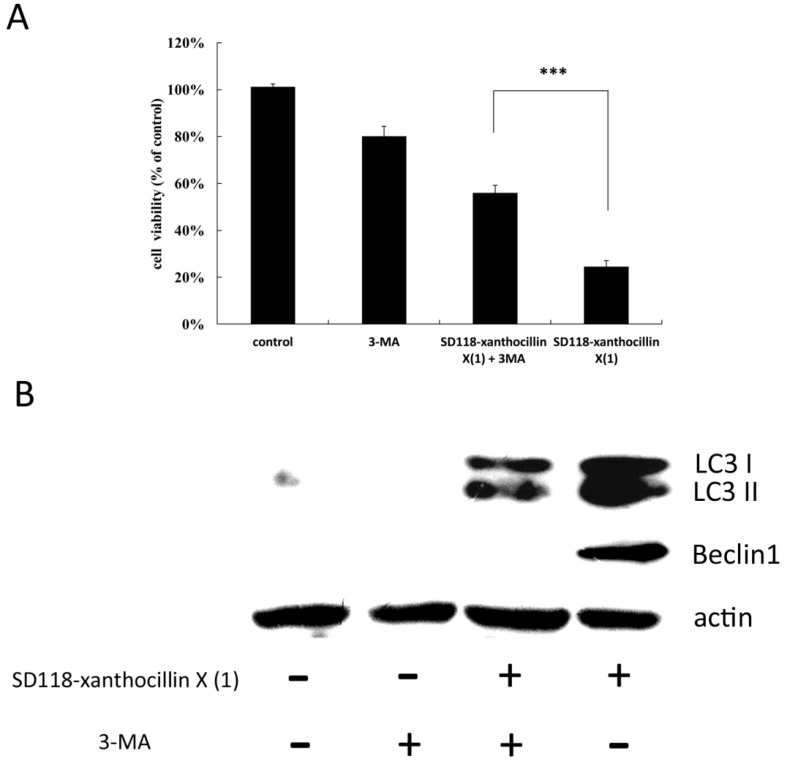
3-MA partially attenuate the autophagic effect of SD118-xanthocillin X (**1**) (**A**) HepG2 cells were treated with 24.3 µM SD118-xanthocillin X (**1**), 5 mM 3-MA or a combination of both for 48 h. Histogram shows the percentage of cell viability. *** indicates a significant difference between SD118-xanthocillin X (**1**) group and 3-MA + SD118-xanthocillin X (**1**) group, by Dunnett’s *t* Test (*** *p* < 0.0001); (**B**) HepG2 cells were treated with 24.3 µM SD118-xanthocillin X (**1**) and 5 mM 3-MA or a combination of both for 48 h. Cell extracts were obtained and underwent western blot analysis, showing conversion of LC3-I to LC3-II and Beclin 1. Protein contents were normalized with β-actin.

To investigate whether inhibition of autophagy would affect the cytotoxicity of SD118-xanthocillin X (**1**), we employed 3-MA, an autophagy inhibitor that acts via the suppression of class III PI3K activity [21], in our cell activity study. The cells were treated with 5 mM 3-MA, and the viability was examined by a cytotoxic activity test. As shown in Figure 5A, the activities of the cells in the control, 3-MA group, SD118-xanthocillin X (**1**) plus 3-MA group and SD118-xanthocillin X (**1**) group were 101 ± 1.3%, 80.18 ± 4.2%, 56.0 ± 3.2% and 24.5 ± 2.6%, respectively (Figure 5A). In comparison to the SD118-xanthocillin X (**1**) plus 3-MA group, the activity of the cells decreased more in the SD118-xanthocillin X (**1**) group. We repeated the same experiments six times, and a similar tendency was detected; the effect was statistically significant. Through western blotting, we confirmed that 3-MA partially attenuated the effect of SD118-xanthocillin X (**1**) on autophagy-related gene expression (Figure 5B). Taken together, our data indicate that SD118-xanthocillin X (**1**) may play a significant role in autophagy-induced cell death.

### 2.5. The Effect of SD118-Xanthocillin X *(**1**)* on Autophagy-Related Signaling Pathways

**Figure 6:**
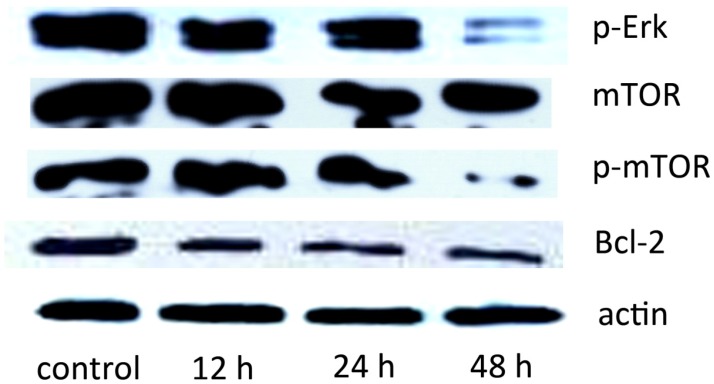
SD118-Xanthocillin X (**1**) is involved in the autophagy signaling pathway. Effect of SD118-xanthocillin X (**1**) on the MEK/ERK/mTOR pathway. HepG2 cells were treated with 24.3 µM SD118-xanthocillin X (**1**), harvested and subjected to western blot analysis, which shows the time-dependent reduction of p-ERK1/2, p-mTOR and Bcl2. Protein contents were normalized with β-actin.

mTOR is a serine/threonine protein kinase that is involved in many pathways in the negative regulation of autophagy, acting as a convergence point [22,23]. In mammalian cells, phosphorylated mTOR (p-mTOR) correlates with the inhibition of autophagy. Rapamycin, an allosteric mTOR inhibitor, has been shown to induce autophagy in malignant glioma cells [24]. To identify the role of SD118-xanthocillin X (**1**) in related pathways, we investigated the mTOR activity by western blotting. As shown in Figure 6, SD118-xanthocillin X (**1**) treatment resulted in decreased levels of p-mTOR in the HepG2 cells, whereas the total mTOR levels were unaffected. It has been previously reported that autophagy is regulated by the MEK/ERK pathway [17]. Therefore, we tested whether p-ERK1/2 levels were regulated by SD118-xanthocillin X (**1**) and found a sustained decrease of p-ERK1/2 in the SD118-xanthocillin X (**1**) group, but not in the control cells. Bcl-2-mediated autophagy occurs through both Beclin 1 and mTOR signaling: Bcl-2 interacts with Beclin 1 and down-regulates autophagy, and it also positively regulates the mTOR signaling pathway, which can inhibit autophagic activity [25,26]. Thus, the above results show that SD118-xanthocillin X (**1**) induces autophagy in HepG2 cells by down-regulating Bcl-2 and inactivating the MEK/ERK pathway (Figure 6).

Previous studies demonstrated that SD118-xanthocillin X (**1**) exhibited effective anti-proliferative activity in the majority of cell lines derived from human solid tumors [15]. In the present study, we provided evidence that this compound slightly induced apoptosis and significantly induced autophagy in HepG2 cells, a process that was followed by autophagic cell death. Autophagy was morphologically and biochemically characterized and included the appearance of autophagic vacuoles in the cytoplasm of the treated HepG2 cells that contained the remnants of organelles, as shown by TEM. We also detected the up-regulated expression of autophagy-related genes and the increased conversion of LC3-I to LC3-II.

We further investigated the signaling pathways affected in SD118-xanthocillin X (**1**)-induced autophagy. Autophagy is positively regulated by the class III PI3K signaling pathway [27,28]. Beclin 1 is a Bcl-2-interacting coiled-coil protein that is a mammalian ortholog of yeast Atg6/Vps30 and is required for the formation of autophagosomes; Beclin 1 is monoallelically deleted in 40%–75% of sporadic breast and ovarian cancers [29]. The overexpression of Beclin 1, which forms a complex with class III PI3K to initiate autophagy, induces cell death in MCF cells [30]. In the present study, we found an increased expression of Beclin 1 and a decreased expression of Bcl-2 in HepG2 cells following SD118-xanthocillin X (**1**) treatment, and this SD118-xanthocillin X (**1**)-induced autophagy in the HepG2 cells was partially attenuated by 3-MA, indicating the involvement of SD118-xanthocillin X (**1**) in drug-induced autophagy.

Autophagy is also negatively regulated by the MEK/ERK signaling pathway [17]. ERKs are a widely conserved family of serine/threonine protein kinases implicated in many cellular processes, such as cell proliferation, differentiation, and apoptosis. ERK1/2 (p42/p44MAPK) can be activated by a great number of oncogenes and extracellular stimuli, including mitogens, growth factors, cytokines, and chemokines. An increasing number of studies have suggested that ERK1/2 plays a role in modulating autophagy [30,31,32,33,34,35] because the constitutive expression of Beclin 1 relies on the basal activity of MEK/ERK, and enhanced Beclin 1 is attributable to an inhibition of MEK/ERK activation. MEK1 and MEK2 activate p44 and p42 through the phosphorylation of activation loop residues Thr202/Tyr204 and Thr185/Tyr187, respectively. Indeed, ERKs require both Thr and Tyr phosphorylation for full activity [36]. Our study showed that SD118-xanthocillin X (**1**) induced autophagy by down-regulating p-Erk1/2 (Thr202/Tyr204 and Thr185/Tyr187) and its activity, which was followed by a reduction in p-mTOR. We found that the non-canonical MEK/ERK module regulates mutually antagonistic processes by regulating Beclin 1 levels. The basal activity of MEK/ERK protected mTOR [17]. As shown in the present study, SD118-xanthocillin X (**1**)-induced autophagy in HepG2 cells involves the inhibition of mTOR phosphorylation. Thus, our data indicated that SD118-xanthocillin X (**1**)-induced autophagy in HepG2 cells is associated with the suppression of the MEK/ERK signaling pathway and the enhancement of class III PI3K/Beclin 1 signaling. This is the first report characterizing the involvement of these signaling pathways in SD118-xanthocillin X (**1**)-induced autophagy. However, one limitation of our study is that we did not examine the upstream effectors of the ERK pathway or other autophagic pathways.

Since the apoptotic and autophagic response machineries share common pathways and cross-talk molecules, such as Bcl-2 and Bcl-xl [37,38], further studies on the signaling pathways of both responses are needed to better clarify the biological role of SD118-xanthocillin X (**1**). These investigations may help uncover the molecular mechanism by which SD118-xanthocillin X (**1**) confer its chemotherapeutic effect in cancer therapy.

## 3. Experimental Section

### 3.1. Test Compound

SD118-Xanthocillin X (**1**) was isolated from a strain of *Penicillium commune* (SD-118) found in a deep-sea sediment. After incubation, the fermented broth was sterilized by adding EtOAc, and the mycelia were separated from the culture broth by filtration. The broth was extracted repeatedly with EtOAc (200 mL each flask) to yield an extract, and the mycelia were air-dried and soaked three times in acetone/water (4:1, v/v) for 3 days. To remove the acetone, the acetone/water extract was evaporated under vacuum and then extracted again with EtOAc to yield another extract. The combined extraction (27 g) was fractionated using silica gel vacuum liquid chromatography (VLC) with petroleum/EtOAc (from 1:0 to 1:1) and CHCl_3_/MeOH (from 20:1 to 0:1) gradient elution to produce 12 fractions (Fr. 1–Fr. 12) [15].

### 3.2. Drug Preparation

SD118-Xanthocillin X (**1**) was dissolved in DMSO and further diluted in PBS. The final solution used in the experiments was 7 g/L; the DMSO concentration was less than 0.1% and did not affect cellular function or the assay systems. 3-Methyladenine (3-MA, Sigma Chemical Co.) was dissolved in 50 °C PBS; upon cooling, the product precipitates out of the solution. The final concentration of 3-MA was 5 mM.

### 3.3. Cell Lines and Growth Inhibition Assay

HepG2 cells were cultured in RPMI 1640 medium and L02 cells were cultured in DMEM medium (HepG2 and L02 cells were purchased from the cell bank of Shanghai Institute of Cell Biology), both of them supplemented with 10% fetal bovine serum and 100 units/mL penicillin. All of the cultured cells were maintained under a humidified 5% CO_2_ atmosphere at 37 °C. The cytotoxic activity of SD118-xanthocillin X (**1**) was determined using a standard MTT, a yellow tetrazole-based colorimetric assay. Briefly, the HepG2 cells were seeded at a density of 7 × 10^3^ cells/well in 96-well microtiter plates. After 24 h, the cells were exposed to different concentrations of the test compounds; cell survival was determined by the addition of MTT solution, as described previously. 

### 3.4. Flow Cytometry Analysis of Apoptosis

Cell apoptosis was examined using an Annexin V-FITC Apoptosis Detection Kit (KeyGEN Biotech, NanJing, China) that measures the externalization of phosphatidylserine in the plasma membrane. The cells were seeded in six-well plates at a density of 5 × 10^6^ cells/mL and incubated overnight; SD118-xanthocillin X (**1**) was added at different time points (12 h, 24 h, and 48 h). The cells were then collected by trypsinization, washed twice with cold PBS and re-suspended in 500 µL of binding buffer (2.5 mM CaCl_2_, 140 mM NaCl and 10 mM Hepes/NaOH, pH 7.4), at a concentration of 1 × 10^6^ cells/mL. Annexin V-FITC and PI were then added to the cells, which were analyzed using flow cytometry. The results were calculated using Cell Quest software and are shown as percentages. Early apoptotic cells were negative for PI and positive for annexin-V, whereas late apoptotic dead cells displayed both high annexin-V and PI labeling [39].

### 3.5. Transmission Electron Microscopy (TEM)

The HepG2 cells were collected after trypsin/EDTA treatment, washed twice with PBS and centrifuged at 800 r/min for 5 min. The cells were then fixed in 3% ice-cold glutaraldehyde for at least 3 h. After washing in cool PBS, the cells were postfixed in osmium tetroxide, and embedded in Spurr’s resin. Thin sections were cut, and double stained with uranyl acetate and lead citrate. Ultrathin sections were observed using a HITACHI H7560 electron microscope. 

### 3.6. Reverse Transcription and Quantitative Real-Time PCR

Total RNA was extracted from cells using the TRIzol reagent (Invitrogen, Carlsbad, NM, USA) following the manufacturer’s protocol. cDNA was synthesized using 1 μg of total RNA with reverse transcriptase and oligo(dT) primers (Fermentas, Burlington, Canada) at 42 °C for 1 h, and the reaction was terminated by heating at 70 °C for 5 min. The mRNA expression levels of beclin 1, LC3 and actin were quantitated using real-time PCR. The cDNA was amplified using SYBR green (TOYOBO, Osaka, Japan) for Beclin 1, LC3 and actin with the Applied Biosystems (ABI7300) Real-Time PCR System primers at an annealing temperature of 60 °C with 1-min annealing time for 45 cycles using primers, as follows: LC3: Forward 5′-AACATGAGCGAGTTGGTCAAGA-3′, Reverse 5′-CTCACCATGCTGTGCTGGTT-3′; Beclin 1: Forward 5′-GAGGGATGGAAGGGTCTAAG-3′, Reverse 5′-TGGGCTGTGGTAAGTAATGG-3′; actin: Forward 5′-CATTGCCGACAGGATGCA-3′, Reverse 5′-GCTCAGGAGGAGCAATGATCTT-3′.

The real-time PCR results were analyzed using the 7300 system software and normalized using the detection of β-actin. The relative expression levels of the target genes were compared to the levels of reference gene (β-actin) using the comparative cycle threshold (ct) method as the fold difference = 2^−(∆ct of target gene − ∆ct of reference)^. 

### 3.7. Western Blotting

Total protein was extracted from cultured cells using the whole protein extraction kit (containing pH 7.4 50 mM Tris, 150 mM NaCl, 1% Triton X-100, 1% sodium deoxycholate, 0.1% SDS and 1 mM PMSF), and the protein concentration was determined using the BCA Protein Assay Reagent (Beyotime, China). Equal amounts of protein were separated discontinuously on 6%–15% SDS-PAGE, transferred to a nitrocellulose membrane, and incubated with anti-human mono/polyclone antibodies overnight at 4 °C. After incubation of the secondary antibody at room temperature for 1 h, the immunoblots were visualized using an enhanced chemiluminescence (ECL) kit. The antibodies used were anti-LC3, anti-Beclin 1, anti-Bcl2, anti-mTOR, phosphorylated-mTOR, phosphorylated-ERK1/2, and anti-actin (Cell Signaling Technology, MA, USA).

### 3.8. Statistical Analysis

The data are reported as the mean ± SD of at least three independent experiments. Each treatment was performed in triplicate culture wells. Statistically significant values were tested by Dunnett’s Tests, and *p* values less than 0.05 were considered statistically significant.

## 4. Conclusion

In summary, the findings presented here indicate that SD118-xanthocillin X (**1**) is highly effective in reducing cell viability and that the reduced survival of HepG2 cells is associated with the initiation of autophagy. SD118-Xanthocillin X (**1**)-induced autophagy resulted from the inhibition of the MEK/ERK-mTOR signaling pathway and the enhancement of the class III PI3K/Beclin 1 signaling pathway.

## References

[1] Naidu S.S., Naidu S. (2010). Studies in Marine Quinone Chemistry.

[2] Nastrucci C., Russo P. (2012). Anticancer drug discovery from the marine environment. Recent Pat. Anticancer Drug Discov..

[3] Jordan M.A., Kamath K., Manna T., Okouneva T., Miller H.P., Davis C., Littlefield B.A., Wilson L. (2005). The primary antimitotic mechanism of action of the synthetic halichondrin E7389 is suppression of microtubule growth. Mol. Cancer Ther..

[4] Okouneva T., Azarenko O., Wilson L., Littlefield B.A., Jordan M.A. (2008). Inhibition of centromere dynamics by eribulin (E7389) during mitotic metaphase. Mol. Cancer Ther..

[5] Kuznetsov G., Towle M.J., Cheng H., Kawamura T., TenDyke K., Liu D., Kishi Y., Yu M.J., Littlefield B.A. (2004). Induction of morphological and biochemical apoptosis following prolonged mitotic blockage by halichondrin B macrocyclic ketone analog E7389. Cancer Res..

[6] Towle M.J., Salvato K.A., Wels B.F., Aalfs K.K., Zheng W., Seletsky B.M., Zhu X., Lewis B.M., Kishi Y., Yu M.J. (2011). Eribulin induces irreversible mitotic blockade: Implications of cell-based pharmacodynamics for *in vivo* efficacy under intermittent dosing conditions. Cancer Res..

[7] Klionsky D.J. (2007). Autophagy: From phenomenology to molecular understanding in less than a decade. Nat. Rev. Mol. Cell Biol..

[8] Rubinsztein D.C., Gestwicki J.E., Murphy L.O., Klionsky D.J. (2007). Potential therapeutic applications of autophagy. Nat. Rev. Drug Discov..

[9] Viola G., Bortolozzi R., Hamel E., Moro S., Brun P., Castagliuolo I., Ferlin M.G., Basso G. (2012). MG-2477, a new tubulin inhibitor, induces autophagy through inhibition of the Akt/mTOR pathway and delayed apoptosis in A549 cells. Biochem. Pharmacol..

[10] Kanzawa T., Germano I., Komata T., Ito H., Kondo Y., Kondo S. (2004). Role of autophagy in temozolomide-induced cytotoxicity for malignant glioma cells. Cell Death Differ..

[11] Laane E., Tamm K.P., Buentke E., Ito K., Khahariza P., Oscarsson J., Corcoran M., Björklund A., Hultenby K., Lundin J. (2009). Cell death induced by dexamethasone in lymphoid leukemia is mediated through initiation of autophagy. Cell Death Differ..

[12] Mizushima N. (2004). Methods for monitoring autophagy. Int. J. Biochem. Cell Biol..

[13] Degenhardt K., Mathew R., Beaudoin B., Bray K., Anderson D., Chen G. (2006). Autophagy promotes tumor cell survival and restricts necrosis, inflammation, and tumorigenesis. Cancer Cell.

[14] Mehrpour M., Esclatine A., Beau I., Codogno P. (2010). Overview of macroautophagy regulation in mammalian cells. Cell Res..

[15] Shang Z., Li X., Meng L., Li C., Gao S., Huang C., Wang B. (2012). Chemical profile of the secondary metabolites produced by a deep-sea sediment-derived fungus Penicillium commune SD-118. Chin. J. Oceanol. Limnol..

[16] Cao Q., Yu C., Xue R., Hsueh W., Pan P., Chen Z., Wang S., McNutt M., Gu J. (2008). Autophagy induced by suberoylanilide hydroxamic acid in Hela S3 cells involves inhibition of protein kinase B and up-regulation of Beclin 1. Int. J. Biochem. Cell Biol..

[17] Wang J., Whiteman M.W., Lian H., Wang G., Singh A., Huang D., Denmark T. (2009). A non-canonical MEK/ERK signaling pathway regulates autophagy via regulating Beclin 1. J. Biol. Chem..

[18] Tanida I., Ueno T., Kominami E. (2004). LC3 conjugation system in mammalian autophagy. Int. J. Biochem. Cell Biol..

[19] Baehrecke E.H. (2005). Autophagy: Dual roles in life and death?. Nat. Rev. Mol. Cell Biol..

[20] Levine B., Deretic V. (2007). Unveiling the roles of autophagy in innate and adaptive immunity. Nat. Rev. Immunol..

[21] Munafó D.B., Colombo M.I. (2001). A novel assay to study autophagy: Regulation of autophagosome vacuole size by amino acid deprivation. J. Cell Sci..

[22] Noda T., Ohsumi Y. (1998). Tor, a phosphatidylinositol kinase homologue, controls autophagy in yeast. J. Biol. Chem..

[23] Wu Y.T., Tan H.L., Shui G., Bauvy C., Huang Q., Wenk M.R., Ong C.N., Codogno P., Shen H.M. (2010). Dual role of 3-methyladenine in modulation of autophagy via different temporal patterns of inhibition on class I and III phosphoinositide 3-kinase. J. Biol. Chem..

[24] Takeuchi H., Kondo Y., Fujiwara K., Kanzawa T., Aoki H., Mills G.B., Kondo S. (2005). Synergistic augmentation of rapamycin-induced autophagy in malignant glioma cells by phosphatidylinositol 3-kinase/protein kinase B inhibitors. Cancer Res..

[25] Pattingre S., Levine B. (2006). Bcl-2 inhibition of autophagy: A new route to cancer?. Cancer Res..

[26] Vara J.Á.F., Casado E., de Castro J., Cejas P., Belda-Iniesta C., González-Barón M.  (2004). PI3K/Akt signalling pathway and cancer. Cancer Treat. Rev..

[27] Petiot A., Ogier-Denis E., Blommaart E.F.C., Meijer A.J., Codogno P. (2000). Distinct classes of phosphatidylinositol 3′-kinases are involved in signaling pathways that control macroautophagy in HT-29 cells. J.Biol. Chem..

[28] Vieira O.V., Botelho R.J., Rameh L., Brachmann S.M., Matsuo T., Davidson H.W., Schreiber A., Backer J.M., Cantley L.C., Grinstein S. (2001). Distinct roles of class I and class III phosphatidylinositol 3-kinases in phagosome formation and maturation. J. Cell Biol..

[29] Liang X.H., Yu J., Brown K., Levine B. (2001). Beclin 1 contains a leucine-rich nuclear export signal that is required for its autophagy and tumor suppressor function. Cancer Res..

[30] Shinojima N., Yokoyama T., Kondo Y., Kondo S. (2007). Roles of the Akt/mTOR/p70S6K and ERK1/2 signaling pathways in curcumin-induced autophagy. Autophagy.

[31] Aoki H., Takada Y., Kondo S., Sawaya R., Aggarwal B.B., Kondo Y. (2007). Evidence that curcumin suppresses the growth of malignant gliomas *in vitro* and *in vivo* through induction of autophagy: Role of Akt and extracellular signal-regulated kinase signaling pathways. Mol. Pharmacol..

[32] Zhu J., Horbinski C., Guo F., Watkins S., Uchiyama Y., Chu C.T. (2007). Regulation of autophagy by extracellular signal-regulated protein kinases during 1-methyl-4-phenylpyridinium-induced cell death. Am. J. Pathol..

[33] Corcelle E., Nebout M., Bekri S., Gauthier N., Hofman P., Poujeol P., Fénichel P., Mograbi B. (2006). Disruption of autophagy at the maturation step by the carcinogen lindane is associated with the sustained mitogen-activated protein kinase/extracellular signal-regulated kinase activity. Cancer Res..

[34] Pattingre S., Bauvy C., Codogno P. (2003). Amino acids interfere with the ERK1/2-dependent control of macroautophagy by controlling the activation of Raf-1 in human colon cancer HT-29 cells. J. Biol. Chem..

[35] Subramaniam S., Unsicker K. (2010). ERK and cell death: ERK1/2 in neuronal death. FEBS J..

[36] Ferrell J.E., Bhatt R.R. (1997). Mechanistic studies of the dual phosphorylation of mitogen-activated protein kinase. J. Biol. Chem..

[37] Maiuri M.C., Zalckvar E., Kimchi A., Kroemer G. (2007). Self-eating and self-killing: Crosstalk between autophagy and apoptosis. Nat. Rev. Mol. Cell Biol..

[38] Zhou F., Yang Y., Xing D. (2011). Bcl-2 and Bcl-xL play important roles in the crosstalk between autophagy and apoptosis. FEBS J..

[39] Deeb D., Gao X., Dulchavsky S.A., Gautam S.C. (2007). CDDO-Me induces apoptosis and inhibits Akt, mTOR and NF-κB signaling proteins in prostate cancer cells. Anticancer Res..

